# Moral and philosophical frameworks for discussing Voluntary Assisted Dying (VAD)

**DOI:** 10.1017/S1478951525000331

**Published:** 2025-04-07

**Authors:** John Attia, Brian Kelly, Megan Best

**Affiliations:** 1 School of Medicine and Public Health, University of Newcastle, Callaghan, NSW, Australia; 2 Department of Palliative and Supportive Care, John Hunter Hospital, Newcastle, NSW, Australia; 3 Psychiatry Liaison Service, John Hunter Hospital, Newcastle, NSW, Australia; 4 Institute for Ethics and Society, University of Notre Dame, Sydney, NSW, Australia

**Keywords:** Ethics, medical assistance in dying (maid), physician-assisted suicide, physician-assisted dying, euthanasia

## Introduction

In Ancient Greece, when treating patients whose condition was incurable, physicians would sometimes use poisons and other techniques, hastening death in order to relieve suffering. The Hippocratic Oath was revolutionary in its time, going against such practices by swearing to “neither give a deadly drug to anybody if asked for it, nor making a suggestion to this effect.” (Verhey 2011) While this has been a foundational text of western medical ethics for over 2000 years, Voluntary Assisted Dying (VAD) has shaken this foundation.

As physicians in Palliative Care, Psychiatry, and Bioethics, we have noticed that many of our colleagues feel ill-equipped to have discussions with patients surrounding VAD; while the pros of VAD are familiar from popular media, the cons are much less so. We believe that doctors need to understand both sides of the argument in order to have balanced and informed discussions with their patients. In this spirit, we offer a reflection on what we have found to be common assumptions when discussing VAD (see summary in Table 1). By necessity, this means exploring the moral and religious perspectives that underpin our medical practice (Sahm 2020).

**Table 1.** Six assumptions around VAD and alternative approaches

Assumption		Alternative
1	A patient’s individual autonomy is the overriding ethical principle in healthcare	Autonomy has limits, should not be confused with independence, and does not remove the need to judge capacity
2	Each person belongs to him/herself and their choice only affects them	An individual’s identity includes his/her social relationships; each person belongs to a community and their choice affects their whole community
3	VAD is part of dying with dignity and helps avoid being a “burden”	The way to honor the patient’s dignity is to celebrate their life and to support them; rather than being a problem, it is an opportunity for others to care for them
4	Life is a burden; each patient has a right to die in the manner and time of their choice	Life is a gift; trying to control death is an illusion and we do better in helping our patients accept this natural end
5	Suffering and pain are completely avoidable	Some suffering is part of being human and we serve our patients better by helping them engage with, rather than escape, their existential distress
6	An option available to animals should be available to humans	Humans are fundamentally different to animals

## Assumption 1: The primacy of patient autonomy

Patient autonomy, the right to make self-governing choices, is often quoted as the rationale for why VAD is warranted (NSW Ministry of Health 2023). However, it is well recognized that this principle is not absolute in healthcare. For example, if a patient with a headache in ED demands an MRI, we do not automatically acquiesce, i.e. we recognize that there are limits to what an autonomous individual can request and that autonomy should not be confused with independence (Quill and Brody 1996). Professional medical ethics demands that the doctor use their expertise to guide patients in their medical decision-making, which includes engaging with the reasons underlying a patient’s choice and judging capacity (Medical Board AHPRA 2020). A patient’s request for VAD is known to be a call for help in the first instance (Chochinov 2004). The will to live, and conversely to die, is known to vary over time (Chochinov et al. 1999) and with intercurrent conditions, e.g. depression (Breitbart et al. 2010).

## Assumption 2: A VAD choice only concerns the individual

Our modern society focuses on the primacy of the individual but Christian Ntizimira, a Rwandan palliative care physician, makes the point that in his culture, the individual is seen as part of their social community; “When you are well, you belong to yourself. But when you are sick, you belong to your family.” (Ntizimira 2023) Seeing the person in isolation ignores the relational aspects of human living. VAD carries not only a documented risk of moral injury to the health care team, but also a more complicated grief and an erosion of community for those left behind (Kelly et al. 2020). The sum of individual acts also shifts the tide of sentiment in the community regarding which lives are worth living. This “slippery slope” can be seen in the Netherlands: while assisted dying was initially highly restricted to adults with life-limiting physical conditions, over time the indications have expanded to mental suffering, to those with longer time frames, and to children (Lemmens 2018), and, in Canada, to those without a terminal diagnosis at all (Gaind 2023).

## Assumption 3: VAD is the only way to retain dignity in dying

VAD is often couched in the language of “dying with dignity.” However, research has shown that what really preserves patient dignity at the end of life is treating them with respect as an individual and upholding their values (Best 2019). There is often a fear of dependency, i.e. not wanting to be a “burden” on others (Monforte-Royo et al. 2011). However, theologian Stanley Hauerwas makes the point that “there is nothing wrong with being a burden!” How we care for those who can no longer care for themselves (e.g. the elderly and the disabled) is one of the ways in which we celebrate the dignity of that person’s life (Hauerwas and Bondi 1976).

On a societal level, Fernandes also warns that judging people as “not of use” was part of the rationale used by physicians during the Nazi regime to justify euthanasia; this label can just as easily be applied by patients to themselves, undermining their own dignity (Fernandes 2020).

## Assumption 4: We have a right to die

VAD advocates often appeal to an individual’s “right” to die, referring to the desire to choose the timing and manner of one’s death but for much of the western medicine’s history, life was seen as a gift from the Creator, and hence the responsibility was to steward this gift wisely and to use the time well. Theologian Stanley Hauerwas puts it this way: “We should view time not as something to be lived through, not life as an end in itself, but rather see life as the gift of time enough for love” (Hauerwas and Bondi 1976). It is not life that is a burden but suffering, and medical care has a responsibility to come alongside and support the patient to live as well as possible (Chochinov 2013).

Experience from overseas indicates that those opting for assisted dying tend to be more educated, more affluent and are essentially seeking more control (Goroncy 2019). Karen Hitchcock, a physician and author, states it this way:

“A good death – an ideal death – is pre-planned, perfectly timed, excretion-free, speedy, neat and controlled. Life is not like this. And yet we think we have a right to ask it of death…. The only way we could come close to meeting all these criteria for a good death would be to put people down when they reach a predetermined age, before the chaos of illness sets in.” (Hitchcock 2016)

This desire for control may spring out of our fear of death, for we seek to control what we fear. Monica Renz, at St-Gallen Hospice in Switzerland, makes the point that our role as physicians is to help our patients face this fear and recognize that none of us are in control of life, much less of death. In helping them reach this acceptance, we affirm that growth and maturation occur throughout life, right up to the point of death (Renz 2015).

Another caveat is that the “right to die” risks becoming the “duty to die”; given the power imbalances between patients at the end of life and their families/carers/clinicians, there can be subtle pressure to end one’s life quickly when one is becoming a “burden” (Meier 2020).

## Assumption 5: Suffering and pain are completely avoidable

While a common argument for VAD is that it is necessary for ending unrelieved pain, physical pain is an uncommon reason for requesting hastened death (Monforte-Royo et al. 2011). Furthermore, VAD requests for anticipated suffering are more common than requests on the basis of current suffering. Early referral of patients to palliative care can alleviate many of the patient’s concerns. Furthermore, modern medicine has perpetuated this idea of freedom from illness and from the limitations of aging, with physicians often persisting with futile treatments right up to the time of death rather than discussing the need to prepare for death (Angus et al. 2004).

The observation that patients at the end of life can reach a peaceful death following engagement with, rather than escape from, existential struggles is one of the reasons why the majority of palliative care societies worldwide are opposed to practices hastening death (Roy and Rapin 1994). Moreover, legalization of VAD has had a negative impact on palliative care resourcing, and we need to continue to advocate for high quality palliative care provision (Jones 2024).

## Assumption 6: We don’t let animals suffer like this

A common refrain in the lay press is that if we put down animals in pain, we should be able to do the same to humans. This is contrary to the idea of the value and dignity of the individual human not only in the Judeo-Christian tradition but also Muslim, Hindu and Buddhist traditions (Grove et al. 2022). This view also downplays the emotional toll, moral distress, cognitive dissonance, and burnout experienced by a majority of the veterinarians who carry out animal euthanasia; the comparison should raise warning bells rather than support for this option (Cooney and Kipperman 2023).

## Conclusion

**Figure 1.**
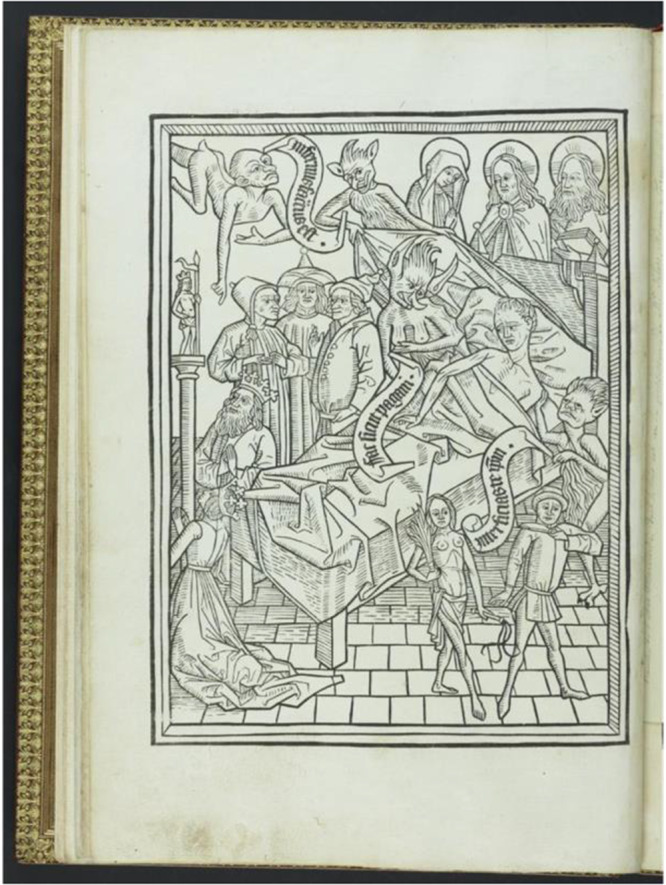
First woodcut from the “Ars Moriendi.” moriens, the dying everyman, lies emaciated in bed, surrounded by 3 physicians discussing his case (middle left). The Virgin Mary, Jesus and God are there at the head of the bed. But there are also demons present. The one in the top left is pointing to hell while his scroll says: hell is prepared for you. The second demon, pulling the bedsheets seems to be impatient to drag him there. Another demon, middle right, touching his shoulder, has a scroll inviting moriens to kill himself, while another man with a knife at his own throat (bottom right), demonstrates one way to do this. (https://www.Loc.Gov/resource/rbc0001.2009rosen0020/?sp=24, used under creative commons license).

It is instructive to go back to the “Ars Moriendi,” the “Art of Dying,” a 15^th^ century book that was essentially a set of instructions for dying well. Originally composed by an anonymous Dominican friar, it was translated into most European languages and was widely read especially in the context of the Black Death. It was later accompanied by woodcuts to serve as visual aids so that those who were illiterate could be taken through these pictures in a process to prepare themselves for death. Despite its age, the “Ars Moriendi” shows remarkable insight into human nature, highlighting a number of temptations to beware of in facing death; the first three are the temptation to lose faith, to despair, and to become impatient (see Figure 1). Indeed, recent work has shown that demoralization – the loss of hope, purpose and meaning in life when one is faced by a stressful situation – is commonly associated with the desire for hastened death in the terminally ill (Robinson et al. 2015). The role of the priests and doctors, seen beside the bed, is to help the dying man hang on to faith, hope, and love (Thornton and Phillips 2009).

It is worth noting that this belief in the value of life is not necessarily rooted in a religious or spiritual belief. Even noted atheist and existentialist Albert Camus wrote: “There is but one philosophical problem and that is suicide…. Even if one does not believe in God, suicide is not legitimate… from the moment when life is accepted as good, it is good for all… In a man’s attachment to life, there is something stronger than all the ills of the world” (quoted in Ashley et al. 2006).
